# A Real-World, Prospective, Multicenter, Single-Arm Observational Study of Duloxetine in Patients With Major Depressive Disorder or Generalized Anxiety Disorder

**DOI:** 10.3389/fpsyt.2021.689143

**Published:** 2021-06-17

**Authors:** Gyorgy Szekeres, Sandor Rozsa, Peter Dome, Gabor Barsony, Xenia Gonda

**Affiliations:** ^1^Department of Psychiatry and Psychotherapy, Semmelweis University, Budapest, Hungary; ^2^Department of Psychiatry, Washington University School of Medicine, St. Louis, MO, United States; ^3^Károli Gáspár University of the Reformed Church in Hungary, Budapest, Hungary; ^4^Nyiro Gyula National Institute of Psychiatry and Addictions, Budapest, Hungary; ^5^Research Flow Kft., Budapest, Hungary

**Keywords:** major depressive disorder, generalized anxiety disorder, duloxetine, health-related quality of life, clinical global impression scale

## Abstract

**Background:** Suboptimal treatment response during anti-depressive treatment is fairly common with the first antidepressant (AD) choice, followed by switching to another agent in the majority of cases. However, the efficacy of this strategy over continuation of the original agent is less solidly documented in real-life studies. The aim of our present study was to ascertain the effects of switching to duloxetine following inadequate response to prior ADs on general illness severity, pain, and health-related quality of life in a large sample of major depressive disorder (MDD) and generalized anxiety disorder (GAD) patients in a prospective, real-world, multicenter, observational study.

**Methods:** A total of 578 participants with MDD or GAD were enrolled in 58 outpatient sites in an 8-week, single-arm, open-label, flexible-dose trial with duloxetine. Severity of symptoms [with Clinical Global Impression-Severity (CGI-S) and Clinical Global Impression-Improvement (CGI-I)], severity of pain (with a Visual Analog Scale), satisfaction with current treatment, and health-related quality of life [with the three-level version of the EuroQol five-dimensional questionnaire (EQ-5D-3L)] measures were recorded at baseline and at follow-up visits 4 and 8 weeks after initiation of treatment. Data were analyzed using ANOVA and mixed linear models.

**Results:** 565 patients completed the study and comprised the analyzed dataset. Results indicated that severity of illness significantly decreased over the 8 weeks of the study and already at 4 weeks in both patient groups. Overall quality of life and all of its subindicators also significantly improved in both patient groups and so did subjective experience of pain. Satisfaction with current treatment also significantly increased during the study period. Frequency of side effects was low. In both GAD and MDD groups, two patients dropped out of the study due to adverse effects, leading to treatment termination in four cases (0.7%).

**Conclusions:** This 8-week, multicenter, flexible-dosing, single-arm, open-label, observational real-life study in MDD and GAD patients switched to duloxetine after inadequate response or low tolerability to other ADs showed a significant positive effect on all outcome measures, including a significant decrease in illness severity as well as significant overall symptomatic improvement, with good tolerability.

## Introduction

Duloxetine is a frequently used psychotropic agent that belongs to the serotonin–norepinephrine reuptake inhibitor (SNRI) type of antidepressants (ADs) (1, 2) and is used to treat both psychiatric [major depressive disorder (MDD) and generalized anxiety disorder (GAD)] and non-psychiatric (e.g., diabetic neuropathic pain, fibromyalgia, stress incontinence) conditions (3, 4). Members of the SNRI family differ from each other in their affinity/selectivity to serotonin and norepinephrine transporters (SERT and NAT, respectively). In this regard, duloxetine has relatively balanced inhibitory properties on these two monoamine transporters. It means that duloxetine has 10-fold greater selectivity for serotonin over norepinephrine reuptake inhibition (the corresponding ratios are about 30 for venlafaxine, 14 for desvenlafaxine, and 1.6 for milnacipran) (1, 5). Duloxetine also has a weak inhibitory effect on dopamine transporters (3), which is also observed in case of venlafaxine when its dose is higher than 300 mg/day (5). This multitarget action mechanism underlies the robust efficacy of duloxetine in the treatment of a wide range of symptoms associated with mood and anxiety disorders and also its pain-relieving effect, which is the most pronounced in the SNRI group and is independent of the presence of depression or anxiety (1, 2, 5, 6).

It is well-known from both clinical studies and everyday practice that suboptimal response (or even non-response) to AD treatment is not uncommon. The STAR^*^D and the European Group for the Study of Resistant Depression trials showed that about 35% of subjects fail to achieve therapeutic response after two consecutive AD trials (7, 8). Several approaches exist to handle insufficient treatment response, from combining a non-pharmacological method [e.g., psychotherapy, electroconvulsive therapy (ECT), physical exercise] with pharmacotherapy (9–11) to various pharmacological strategies, including increasing the dose of the originally administered AD (“dose maximizing”) (10), adding a second non-AD-type drug (e.g., lithium) to the already administered AD (“augmentation”), combining the already administered AD with another AD (“combination”), and changing the already administered AD to a novel one (“switching”) (10). However, the efficacy of the above strategies, and specifically of switching, is underinvestigated in real-life setting using prospective, open-label, observational studies in large patient samples in general and especially in the case of duloxetine.

The objective of the current study was to ascertain the effects of the commencement of duloxetine treatment on general illness severity, symptomatic improvement, pain, and health-related quality of life in a real-world observational study among patients with MDD or GAD without adequate response to and/or with remarkable side effects during treatment with a prior AD.

## Materials and Methods

### Participants and Study Design

**Figure 1:**
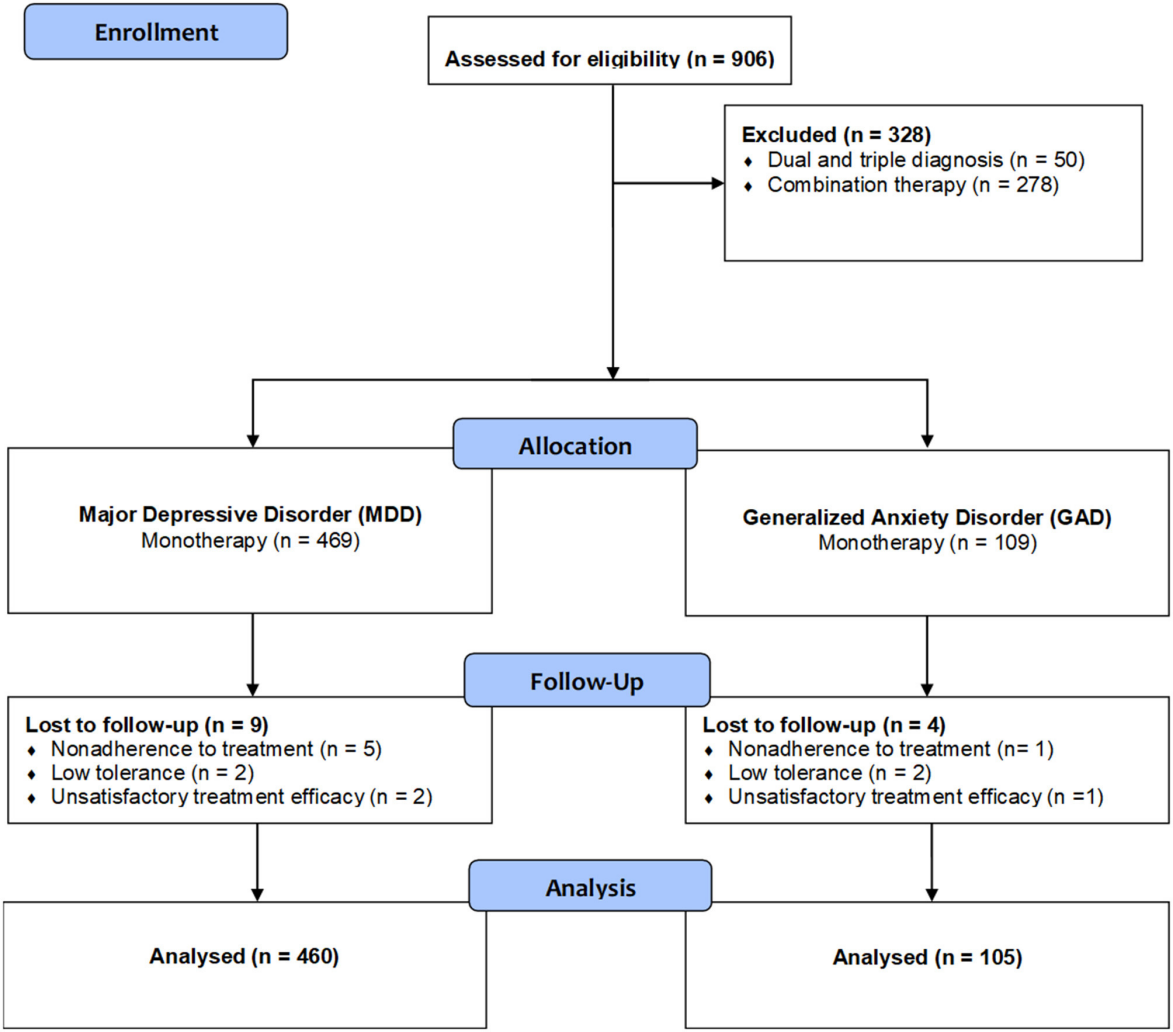
Flowchart of the progression of participants through the study.

Of the initially recruited 906 patients at 65 sites for the investigation of switching to duloxetine involving several neuropsychiatric diagnoses, 578 eligible participants with MDD and GAD were enrolled in a multicenter, 8-week, single-arm, open-label, flexible-dose trial with duloxetine, conducted at psychiatric clinical practices in Hungary. Sites included only outpatient providers, such as outpatient psychiatric practices or outpatient units of psychiatric departments of hospitals. Only specialist psychiatric settings participated as centers; no general practice settings were involved. Number of patients per site ranged between 1 and 30; average number of patients per site was 9.1. Of the originally involved 65 centers, patients from 58 sites were included in the present analysis; patients for the remaining six sites were excluded either for having only diagnoses other than MDD or GAD, multiple diagnoses, multiple previous medications, terminated the study before the final visit or were lost to follow-up. Subjects were recruited through clinician referrals. Inclusion criteria included age ≥18 years, current diagnoses of MDD or GAD, determined on the basis of a psychiatrist-conducted clinical interview to verify the presence of the Diagnostic and Statistical Manual of Mental Disorders, Fifth Edition (DSM-5) criteria, and written informed consent. Psychiatric exclusion criteria included diagnoses of schizophrenia or other psychotic disorders, dementia or other cognitive impairment, and drug or alcohol abuse or dependence within the previous 6 months based on medical records, unstable general medical conditions, and lack of capacity to consent to study participation based on evaluation at inclusion. Patients where duloxetine administration was contraindicated for psychiatric and somatic reasons as stated in the Summary of product characteristics (SPC), including those concomitantly taking non-selective irreversible monoamine oxidase (MAO) inhibitors or cytochrome P450 (CYP)1A2 inhibitors fluvoxamine, ciprofloxacin, or enoxacin, presenting with comorbid hepatic impairment or severe liver disease, severe kidney impairment (creatinine clearance <30 ml/min), or comorbid unstable hypertonia possibly triggering hypertensive crisis, were also excluded from the study. For the present analysis, we also excluded those patients who had more than one psychiatric diagnosis and who were comedicated with any other psychiatric medication except for benzodiazepines following initiation of duloxetine treatment. Figure 1 contains an attrition flow diagram that visualizes the inclusion and exclusion process. Participants had the right to withdraw at any point during treatment. The study protocol was reviewed by the National Scientific and Ethical Committee of Hungary (Reference No. 60678/2017/EKU). All patients signed an informed consent form before collection of any data. Study visits were at baseline (visit 1) and at 4 and 8 weeks (visits 2 and 3, respectively) at which time the treating psychiatrist administered clinical rating scales. Safety measures recorded at every visit included spontaneously reported treatment-emergent adverse events.

The initial dose of duloxetine was 30, 60, 90, or 120 mg/day, and the dose was increased according to the clinical response and patient tolerance (the dose was not fixed), and the maximum dose was 120 mg/day.

### Measures

Study assessments included a standardized evaluation of demographic (age, gender) and clinical characteristics as well as Clinical Global Impression-Severity (CGI-S) at baseline and CGI-S and Clinical Global Impression-Improvement (CGI-I) scales (12), Visual Analog Scale (VAS) for pain (13), satisfaction with current treatment, and the three-level version of the EuroQol five-dimensional questionnaire (EQ-5D-3L) at the two follow-up visits 4 and 8 weeks after initiation of treatment. All assessments were completed by board-certified psychiatrists.

CGI-S is a 7-point scale where the clinician rates the severity of the patient's illness at the time of assessment. Considering total clinical impression, a patient is assessed on the severity of mental illness at the time of rating and rated as follows: 1, normal, not at all ill; 2, borderline mentally ill; 3, mildly ill; 4, moderately ill; 5, markedly ill; 6, severely ill; or 7, extremely ill. Besides severity values, CGI-S was also used to estimate remission, where patients rated 1 or 2 were considered to be in remission, while patients rated 3–7 were considered to be non-remitters.

CGI-I is a 7-point scale where the clinician assesses improvement or worsening of illness severity compared to baseline as follows: 1, very much improved; 2, much improved; 3, minimally improved; 4, no change; 5, minimally worse; 6, much worse; 7, very much worse.

Subjective experience and severity of pain in the present study was evaluated by a VAS used for digitizing values that cannot be numerically measured, which started with 0 for “no pain” and ended with 10 for “very severe pain” and leaving 1 cm between values giving a numeric value to every centimeter.

Satisfaction with current treatment was rated on an 11-point Likert scale ranging from 0 = “not at all satisfied” to 10 = “very satisfied.”

Health status was measured using EQ-5D-3L, which asks respondents to describe their health using five domains (mobility, self-care, usual activities, pain/discomfort, and depression/anxiety). In each domain, respondents indicate whether they have 1-no problems, 2-some problems, or 3-extreme problems on the day of EQ-5D-3L completion, which were used in calculations involving the individual domains as numerical values. The health status estimates for the total score were derived from the UK algorithm in this study to produce values between 0 and 1, where 1 represents “full health” and 0 represents death (14).

### Statistical Methods

Demographic data were examined using independent-samples *t*-tests and Fisher's exact tests for dichotomous variables. Independent-samples *t*-tests and repeated-measures ANOVAs were employed to compare changes in psychometric ratings. Differences between outcome variables at different time points were also analyzed by a linear mixed model. The time factor for the model was set at baseline and weeks 4 and 8. *Post-hoc* pairwise comparisons were carried out using paired *t*-test. Linear regression analyses were carried out to determine the effects of dosing. Chi-square tests were used to compare the number of patients in remission in the two clinical groups at the three visits. Statistical significance was accepted for p-values < 0.05. Only data from patients completing the study were analyzed. The statistical analysis was performed using SPSS version 22.0 (IBM SPSS Statistics, Chicago, IL, USA).

## Results

### Participant Flow

A total of 578 participants were included in the study and assigned to two groups: 469 participants to the MDD and 109 to the GAD. Figure 1 shows the progression of participants throughout the study. In the MDD group, nine participants, and in the GAD group, four participants were lost to follow-up due to non-adherence to treatment, low tolerance, or unsatisfactory treatment efficacy. Finally, 565 participants with MDD or GAD aged 20–91 years were included in the final analysis. The sociodemographic data of the samples are summarized in Table 1.

**Table 1:** Characteristics of patients who completed 8 weeks of treatment.

**Characteristics**	**MDD**		**GAD**	
	***n***	**%**	***n***	**%**
Number of patients	460	-	105	-
**Sex**				
Male	132	29	44	42
Female	328	71	61	58
**Age groups**				
18–35	37	8	20	19
36–60	270	59	61	58
61–90	153	33	24	23

*MDD, major depressive disorder; GAD, generalized anxiety disorder*.

### Medication Used Prior to Switching to Duloxetine Treatment

In the MDD group, prior to initiating duloxetine at visit 1, 1.2% of the patients were treated with a tricyclic antidepressant (TCA), 51.7% with a selective serotonin reuptake inhibitor (SSRI), 12.9% with an SNRI other than duloxetine, 2.4% with a melatonin MT1/MT2 agonist, 6.1% with an α2 antagonist (mirtazapine), 3.4% with a selective serotonin reuptake enhancer (SSRE), and 0.2% with a norepinephrine–dopamine reuptake inhibitor (NDRI), or reversible inhibitor of monoamine oxidase (RIMA). The rest of the patients received other medications (including mood stabilizers, antipsychotics, or anxiolytics). In the GAD group, 1.0% were taking a TCA, 42.7% an SSRI, 9.4% an SNRI other than duloxetine, 7.1% mirtazapine, 1% an SSRE, and the rest an anxiolytic, mood stabilizer, or an antipsychotic before initiation of duloxetine treatment.

### Duloxetine Dosing and Dose Adjustment During the Study Visits in the Analyzed Dataset of Patients Completing the Study

The initial dose of duloxetine was 30, 60, 90, or 120 mg/day, and the dose was increased according to clinical response and patient tolerance (the dose was not fixed), and the maximum dose was 120 mg/day.

At the first visit, in the MDD group, the starting dose was 30 mg in 30.65%, 60 mg in 45.44%, 90 mg in 23.26%, and 120 mg in 0.65% of the patients, while in the GAD group, initial duloxetine dose was 30 mg in 42.86%, 60 mg in 46.67%, 90 mg in 10.48%, and 120 mg in 0.95% of the patients. The most common starting dose was 60 mg in both groups (Table 2).

**Table 2:** Doses of duloxetine and dose adjustments during the 8-week study period in MDD and GAD patients.

	**Visit 1**		**Visit 2**		**Visit 3**	
	**MDD**	**GAD**	**MDD**	**GAD**	**MDD**	**GAD**
*N*	460	105	460	105	460	105
Minimum dose, mg	30	30	30	30	30	30
Maximum dose, mg	120	120	120	120	120	120
Mean dose, mg	58.17	50.86	71.15	66.57	74.74	70.57
Median dose, mg	60	60	60	60	90	60
Dose increased from previous visit *N* (%)			185 (40.22%)	53 (50.48%)	58 (12.61%)	15 (14.29%)
Dose continued from previous visit *N* (%)			274 (59.56%)	52 (49.52%)	398 (86.52%)	89 (84.76%)
Dose decreased from previous visit *N* (%)			1 (0.22%)	0 (0%)	4 (0.87%)	1 (0.95%)
Mean dose increase from previous visit mg			32.43	31.13	30.52	30
Mean dose decrease from previous visit mg			30	0	30	30
30 mg, *N* (%)	141 (30.65%)	45 (42.86%)	44 (9.57%)	15 (14.29%)	42 (9.13%)	15 (14.29%)
60 mg, *N* (%)	209 (45.44%)	48 (46.67%)	212 (46.09%)	54 (51.43%)	168 (36.52%)	41 (39.05%)
90 mg, *N* (%)	107 (23.26%)	11 (10.48%)	193 (41.96%)	34 (32.38%)	232 (50.43%)	46 (43-81%)
120 mg, *N* (%)	3 (0.65%)	1 (0.95'%)	11 (2.39%)	2 (1.90%)	18 (3.91%)	3 (2.86%)

*MDD, major depressive disorder; GAD, generalized anxiety disorder; N, number of patients; %, percentage of patients*.

During the second visit at 4 weeks, in the MDD group, 59.56% of patients continued their dose, the dose was increased in 40.22%, and one patient (0.22%) had dose reduction. In the GAD group, 49.52% of patients continued their initial dose, 50.48% had dose increase, and no patient had dose decrease. The mean dose at the second visit was 71.15 mg in the MDD group and 66.57 mg in the GAD group (Table 2).

During the third visit at 8 weeks, in the MDD group, 86.52% continued their dose from the previous visit, 12.1% had dose increase, and 0.87% of patients had dose decrease. In the GAD group, 84.76% of patients continued their previous dose, 14.29% had dose increase, and 0.95% had dose decrease. The mean doses at the third visit were 74.74 mg in the MDD group and 70.57 mg in the GAD group. The median dose was 60 mg in all visits in both patient groups except at the third visit wherein the median dose was 90 mg in the MDD group (Table 2).

### Efficacy Evaluation

Severity of illness in both clinical groups significantly decreased over the 8-week study period according to CGI-S score (*p* < 0.001 in both groups; see Table 3). Parallel with this, a significant improvement in illness severity over the study period was also observable according to the CGI-I scale in both groups (*p* < 0.001 in both groups; see Table 3). Bonferroni *post-hoc* tests revealed that all differences were statistically significant, both from baseline to visit 2 at 4 weeks and from visit 2 at 4 weeks to visit 3 at 8 weeks.

**Table 3:** Outcome measures for patients treated with duloxetine and effects of dosing.

	**Baseline**		**4 weeks**		**8 weeks**		**Over time variation *p*-value**	**Effects of dosing (Baseline vs. 8 weeks)**
	***Mean***	***SD***	***Mean***	***SD***	***Mean***	***SD***		
**GAD (*****N*** **=** **105)**								
Clinical global impression								
Severity (CGI-S)	4.38	0.92	3.35	1.08	2.50	1.15	<0.001	β = −0.21 (*p* = 0.024)
Improvement (CGI-I)	-	-	2.55	0.83	1.83	0.84	<0.001	-
Subjective experience of pain (VAS)	3.17	3.02	2.07	2.46	1.57	2.36	<0.001	n.s.
Satisfaction with current treatment	4.07	2.08	6.93	2.33	8.36	2.24	<0.001	n.s.
EQ-5D-3L	0.55	0.20	0.72	0.18	0.84	0.16	<0.001	n.s.
Mobility	1,27	0,44	1,14	0,35	1,09	0,28	<0.001	-
Self-care	1.22	0.42	1.10	0.30	1.07	0.25	<0.001	-
Usual activities	1.66	0.60	1.35	0.52	1.15	0.36	<0.001	-
Pain/discomfort	1.86	0.60	1.57	0.59	1.36	0.50	<0.001	-
Anxiety/depression	2.40	0.49	2.09	0.28	2.02	0.14	<0.001	-
**MDD (*****N*** **=** **460)**								
Clinical global impression								
Severity (CGI-S)	4.71	0.83	3.63	1.01	2.72	1.12	<0.001	β = −0.10 (*p* = 0.032)
Improvement (CGI-I)	-	-	2.56	0.74	1.85	0.76	<0.001	-
Subjective experience of pain (VAS)	4.92	3.08	3.04	2.46	1.86	1.91	<0.001	n.s.
Satisfaction with current treatment	4.11	2.10	7.16	2.12	8.27	2.18	<0.001	β = 0.13 (*p* = 0.007)
EQ-5D-3L	0.45	0.21	0.68	0.19	0.81	0.17	<0.001	β = 0.17 (*p* = 0.000)
Mobility	1.45	0.51	1.30	0.46	1.22	0.42	<0.001	-
Self-care	1.32	0.49	1.17	0.39	1.04	0.29	<0.001	-
Usual activities	1.90	0.64	1.44	0.56	1.22	0.43	<0.001	-
Pain/discomfort	2.21	0.62	1.75	0.53	1.48	0.43	<0.001	-
Anxiety/depression	2.48	0.50	2.10	0.30	2-01	0.10	<0.001	-

*MDD, major depressive disorder; GAD, generalized anxiety disorder; CGI-S, Clinical Global Impression-Severity; CGI-I, Clinical Global Impression-Improvement; VAS, Visual Analog Scale; EQ-5D-3L, three-level version of the EuroQol five-dimensional questionnaire*.

When considering those with CGI-S score <3 to be in remission, in case of MDD, 0.43, 12.83, and 41.3% of patients were in remission at baseline (patients in remission at baseline were switched to duloxetine due to tolerability problems), 4 and 8 weeks, respectively [chi-square (2,460) = 268.3042, *p* < 0.00001]. In case of GAD patients, 0.95, 19.05, and 54.28% of patients achieved remission at baseline, 4 and 8 weeks of treatment, respectively [chi-square (2,105) = 82.9163, *p* < 0.00001].

Linear regression analysis showed a significant effect of dose on changes in CGI-S in GAD and MDD patients (*p* = 0.024 and *p* = 0.032 for GAD and MDD groups, respectively; see Table 3). Age and sex were not significant covariates, except of CGI-S in GAD.

### Health Status and Subjective Experience of Pain

Overall health status, as measured by EQ-5D-3L, as well as all five of its status indicators (mobility, self-care, usual activities, pain/discomfort, anxiety/depression) also significantly showed improvement with time during the 8 weeks of duloxetine therapy in both groups (*p* < 0.001 in both groups; see Table 3). A significant improvement over time during the whole study period was also observable for subjective experience of pain in both groups (*p* < 0.001; see Table 2). Bonferroni *post-hoc* tests revealed that all differences were statistically significant both from baseline to visit 2 at 4 weeks and from visit 2 at 4 weeks to visit 3 at 8 weeks, except for no significant differences between the second and third visits in the GAD group for EQ-5D-3L for mobility (*p* = 0.1863) and self-care (*p* = 0.4380).

Linear regression analysis showed a significant effect of dose only in the MDD group on EQ-5D-3l total scores (*p* < 0.0001). Age and sex were not significant covariates.

### Satisfaction With Treatment

During the whole 8-week study period, satisfaction with treatment significantly increased in both groups over the 8-week study period (*p* < 0.001 in both groups; see Table 3) and from visit 1 to visit 2 and from visit 2 to visit 3 according to Bonferroni *post-hoc* tests (*p* < 0.001 for both groups and both cases).

Linear regression analysis showed a significant effect of initial dose on satisfaction with current treatment only in the MDD group (*p* = 0.007; see Table 3). Age and sex were not significant covariates.

### Adverse Effects

In the case of the GAD group, of the initially included 109 patients, two (1.83%) patients reported adverse effects at the second visit at 4 weeks. In one case, there was verified association, and in the other, possible association with duloxetine treatment. In the case of one patient, the side effects were mild and did not require intervention, while in the other case, the reported side effect was severe and led to termination of duloxetine treatment at 4 weeks (0.92%). At the third visit, after 8 weeks of treatment, one (0.93%) patient reported moderately severe side effects likely related to duloxetine, leading to termination of treatment in the GAD group. Altogether, during the 8 weeks of the study, of the 109 included patients, three reported adverse effects of different severities (2.75%) and two had to terminate treatment (1.83%).

In the case of MDD, of the initially involved 464 patients, at the second visit after 4 weeks, three patients (0.65%) reported adverse effects, with possible, likely, and verified association with duloxetine. Side effects were mild in two cases not requiring intervention and moderately severe in one case where duloxetine treatment was terminated (0.22%). At the third visit, one patient reported a mild adverse effect likely related to duloxetine, which led to treatment termination at 8 weeks (0.22%). Altogether, during the 8-week study period in the MDD group, four patients reported adverse effects (0.86%), which led to treatment termination in two cases (0.43%).

## Discussion

In our present 8-week, multicenter, flexible-dosing, single-arm, open-label, observational real-life study in MDD and GAD patients switched to duloxetine after inadequate response or tolerability to other ADs, we observed a significant positive effect on all outcome measures, including a significant decrease in illness severity as well as significant overall symptomatic improvement. By the end of the 8-week treatment, 41.3% of MDD and 54.28% of GAD patients previously non-responding to AD treatment and switched to duloxetine reached remission, as indicated by a score <2 on the CGI-S. Furthermore, a significant improvement in all self-rated health status dimensions including mobility, self-care, usual activities, pain/discomfort, or anxiety/depression and significant improvement of subjective experience of pain were observable in the case of both diagnoses. A steady and significant improvement of satisfaction with treatment was also observable during the study. Finally, a significant effect of initial treatment dose was also observed for change in severity of overall symptoms in both groups, with a larger decrease for a higher initial dose, and for satisfaction with treatment and health status in GAD patients, where a larger initial dose was associated with higher satisfaction and more improved health status. Overall, our results support beneficial effects of switching to duloxetine in patients not responding to other conventional and newer-generation ADs in MDD or GAD or who had intolerable side effects from their previous AD.

Duloxetine has been approved for the treatment of various psychiatric diagnoses including MDD and GAD. Especially in the case of MDD, there is a significant proportion of patients not adequately responding to first treatment trials, and ~35% can be considered treatment resistant (15) as not responding to the first two trials of adequate ADs. In the case of GAD, while SSRIs and SNRIs are recommended as first-line treatment, there is no relevant clinical evidence choosing between the medications and up to half of patients do not respond adequately to treatment (16). Thus, switching treatment due to lack of efficacy or suboptimal tolerability is a frequent clinical practice. Finding the right treatment in several cases requires several attempts and trials, which significantly extends the suffering of patients and leads to increased economic costs in terms of both treatment and loss of functionality.

### Efficacy of Switching to Duloxetine Treatment Over 8 Weeks in Major Depressive Disorder and Generalized Anxiety Disorder Patients

In our observational study, the significant overall improvement, symptomatic decrease, and increase in proportion of remitted patients signify that duloxetine may be a beneficial choice for MDD and GAD patients requiring a switch due to non-response to other treatments. In our 8-week trial, not only did we observe a significant improvement along all measures by completion of the trial but also the improvement was observable in severity measures already at the fourth week of treatment in both MDD and GAD patients. Further significant improvement continued all through the second follow-up visit, 8 weeks after initiation of switch, where 41.3% of MDD and 54.28% of GAD patients reached remission corresponding to a CGI-S score <2, which is in line with previous results and models. Following acute treatment with ADs in general, ~37% of MDD patients reach remission by week 9 [based on a Patient Health Questionnaire (PHQ)-9 score of 4 or less] (17), while in the case of acute duloxetine treatment, remission rates are reported between 44 and 55% in placebo-controlled studies using various instruments (18). In the case of GAD, approximately one-third to one-half of patients achieve remission with 8–9 weeks of acute treatment with ADs in general (19), while remission rates between 31 and 38% have been reported in acute 60–120-mg duloxetine treatment of GAD patients in double-blind, placebo-controlled studies (20). It must be noted that in our study, we investigated patients switched from other ADs mostly due to inadequate response. In a network meta-analysis of eight ADs for the treatment of GAD involving placebo-controlled and head-to-head comparison studies (21), duloxetine in comparison to placebo was associated with the highest change of symptoms from baseline (with a mean difference of −3.0, 95% CI: −4 to −2.2) and had the second highest response rate [odds ratio (OR): 2.1, 95% CI: 1.6–2.2] after venlafaxine (21). For MDD patients in a similar network meta-analysis with 21 ADs for acute treatment (22), duloxetine was the third most efficacious drug in terms of response rates after amitriptyline and mirtazapine with an OR of 1.85 (95% CI: 1.66–2.07). While our CGI-S-based remission rate for depression is in the lower range of those observed for duloxetine in previous studies, it must be noted that our patients were non-responders to at least one previous treatment, so this population may contain a higher than usual proportion of treatment-resistant depressed patients. On the other hand, in the case of GAD patients, remission rates (based on CGI-S) in the present study were significantly higher than figures reported previously in non-switched patients, possibly suggesting that the second choice of treatment in those patients who do not respond to initial therapy leads to remission in a larger portion of patients in the case of GAD.

### Effects of Switching to Duloxetine on Functionality-Related Health Status in Major Depressive Disorder and Generalized Anxiety Disorder Patients

Notably, beyond significantly decreasing symptom severity, functionality-related health status measures such as self-care, pursuing of usual activities, and mobility also showed significant improvement in both groups already at the fourth week of treatment with an overall significant increase in EQ-5D-3L in both groups and with further increase from the fourth week to the eighth week in the total score as well as all sub-measures in the MDD group. However, in GAD patients, no further significant improvement in mobility and self-care was observable beyond week 4 of duloxetine treatment. Furthermore, a significant effect of dose was observed on improvement on EQ-5D-3L total score only in the MDD group, with bigger improvement for higher doses.

### Effects of Switching to Duloxetine Treatment on Subjective Experience of Pain in Generalized Anxiety Disorder and Major Depressive Disorder Patients

As pain is a frequent but often overlooked symptom of depression and anxiety disorders (23, 24) that often persists as a residual symptom in spite of treatment significantly impairing quality of life (25, 26), we also looked at the effect of switching to duloxetine treatment on subjective evaluation of pain. In both MDD and GAD patients, subjective ratings of pain were significantly reduced during the 8-week study period according to both the VAS evaluation and the pain/discomfort subscale of EQ-5D-3L in both groups. Along both measures of pain, significant effect in both groups appeared already in the fourth week of treatment and further significant improvement continued through the eighth week. Notably, there was no significant dose effect on pain measurement by VAS in either the MDD or the GAD groups. In previous double-blind, placebo-controlled acute treatment studies of duloxetine in MDD, a significant improvement in painful physical symptoms was also observed (27) and often as early as the second week of the study (18). Furthermore, in previous double-blind, placebo-controlled studies in the acute treatment of GAD, duloxetine was also significantly effective in improving subjective feelings of pain (20). Thus, our open-label naturalistic study in a clinical setting confirms previous findings on the beneficial effects of duloxetine on pain associated with affective disorders.

### Satisfaction With Treatment During the 8-Week Duloxetine Treatment Period

As satisfaction with treatment is a central factor determining treatment adherence, compliance, and persistence (28), we specifically looked at self-reports of patients on satisfaction with their current duloxetine treatment. Satisfaction significantly improved with time in both MDD and GAD groups; furthermore, a significant effect of dose was observable only for the MDD group but not the GAD group with higher satisfaction with higher doses. Besides the significant increase, the fact that at the third visit (8 weeks), in GAD patients, the mean score of satisfaction was 8.36 and, in MDD patients, 8.27 from a maximum of 10 indicates good acceptability of duloxetine in our patient populations.

### Adverse Reactions During 8 Weeks of Switching to Duloxetine Treatment

The very low numbers of both GAD and MDD patients who reported adverse effects (2.75 and 0.86% in the GAD and MDD groups, respectively) and had to terminate treatment due to these adverse reactions (1.83 and 0.43% in the GAD and MDD groups, respectively) are much lower than the 12–14% adverse event-related discontinuation rates reported in previous double-blind, placebo-controlled studies for MDD (18, 29–31) and the 5% of adverse events in double-blind, placebo-controlled acute duloxetine treatment studies of GAD (20). In GAD patients, in a network meta-analysis of eight ADs for acute treatment, although duloxetine was found to be effective, its acceptability and tolerability were found to be lower than placebo and other drugs such as venlafaxine and escitalopram (21). In a similar network meta-analysis of 21 ADs in the acute treatment of MDD, duloxetine was among the medications with the highest dropout rates as one of the least tolerable drugs, with worse tolerability compared to several other drugs including escitalopram, fluoxetine, paroxetine, sertraline, and vortioxetine (22). Thus, our real-life results in a naturalistic clinical setting suggest that duloxetine may be significantly better tolerated in both MDD and GAD patients than suggested by double-blind, placebo-controlled trials.

### Strengths and Limitations

Several limitations of our study must be noted. First of all, there was no placebo or active comparator treatment, which limits our capacity to draw reliable conclusions regarding efficacy. However, such single–arm, open-label studies combine benefits of drug and placebo response (29) with more favorable remission rates than those seen in placebo–controlled, double-blind trials. Also, in the lack of comparators, outcome measures can only be compared to baseline values. At the same time, an open–label, single-arm study more closely resembles standard clinical practice than double-blind, placebo-controlled designs, providing the possibility for more realistic observations especially given the large sample size employed in our present analysis (29). A further shortcoming of our study is that diagnoses were ascertained by clinical interviews administered by a psychiatrist but not structured instruments, and scales for the assessment of specific depressive and anxious symptoms were not used. Accordingly, we were unable to ascertain the effect of duloxetine treatment on the given symptoms of MDD and GAD. The final limitation is the relatively short 8-week assessment period; a longer study period would have allowed for the more precise observation of later emerging adverse effects as well as relapse rates.

## Conclusion

Overall, results of our naturalistic, observational study in a large population of MDD and GAD patients indicate beneficial effects of duloxetine on illness severity, functional outcomes, and subjective experience of pain. We also found good satisfaction with treatment and good tolerability in both GAD and MDD groups and even in patients who had to switch treatment due to lack of response to other ADs.

## Data Availability Statement

The raw data supporting the conclusions of this article will be made available by the authors, without undue reservation.

## Ethics Statement

The studies involving human participants were reviewed and approved by National Scientific and Research Ethical Committee of Hungary (Reference No. 60678/2017/EKU). The patients/participants provided their written informed consent to participate in this study.

## Author Contributions

GS and GB contributed to the conceptualization and methodology. SR and XG contributed to the statistical analysis. PD, XG, and SR contributed to writing the original draft preparation and review and editing. All authors read and approved the final manuscript.

## Conflict of Interest

GB is employed by Research Flow Kft. This research was supported by Krka. The funders had no role in the design of the study; in the collection, analyses, or interpretation of data; in the writing of the manuscript, or in the decision to publish the results.
